# The neural correlates of interference control dysfunction of working memory in major depressive disorder: An event-related potential study

**DOI:** 10.3389/fpsyt.2022.1003491

**Published:** 2022-09-30

**Authors:** Sha-yu Jin, Jia-zhao Zhang, Ru-hong Sun, Chen-guang Jiang, Jun Wang, Zhen-he Zhou

**Affiliations:** ^1^Department of Psychiatry, The Affiliated Wuxi Mental Health Center of Nanjing Medical University, Wuxi, Jiangsu, China; ^2^3 Grade 2019 Class 6, Basic Medicine College of Jinzhou Medical University, Jinzhou, Liaoning, China

**Keywords:** major depressive disorder, interference control, working memory, event-related potentials, Brown-Peterson task

## Abstract

Interference control function is a key function in a series of specific functions of working memory (WM), which is usually impaired in patients with major depressive disorder (MDD). Event-related potentials (ERPs) have advantages in exploring the neural processing of interference control and WM impairment, and therefore, it is helpful to further understand the neural mechanism of MDD. In the present study, 44 patients with MDD and 44 age- and sex-matched healthy controls (HCs) were recruited. All participants completed a 4-gradient difficulty Brown-Peterson task (BPT), whose difficulty was manipulated by changing the demand of interspersed distraction tasks. High-density EEG was simultaneously recorded. The hit rate and reaction time (RT) toward the target stimulus as well as the underlying ERP features were analyzed. The results showed that, when compared with HCs, MDD patients had significantly lower hit rates and longer RTs among all four difficulties of BPT. For ERP components, no significant between-group difference was found in either N100 or P200 average amplitudes; however, the centroparietal late positive potential (LPP) amplitude of both MDD group and HC group decreased with the increase of BPT difficulty, despite the pattern of the HC group was relative moderate. For both groups, the LPP amplitude was significantly smaller in high-order difficult BPT tasks than in low-order difficult tasks. Moreover, LPP amplitude in high-order difficult tasks was much smaller in MDD group than that of HC group. Our findings suggest that failure to control interference well may play a critical role in the impairment of WM in patients with MDD, and provided new evidence that the neural correlates of interference control dysfunction of WM in MDD.

## Introduction

Major depressive disorder (MDD) is a kind of mental disorder characterized by low emotion, diminished interest, and loss of happiness (1). It is one of the most common mental disorders, resulting in a sharp decline in quality of life and a significant personal and economic burden. MDD patients have a greater decline in thinking, attention, and especially memory than before the disease, and this involves changes in cognitive control in MDD (2, 3). It has been proposed that impaired working memory (WM) function is a sign of cognitive control deficits in MDD (4).

Working memory refers to a system with limited capacity (5, 6), which is used to temporarily maintain and store information (7). It is a basic support structure of the thinking process (8). WM includes the process of inhibition, transfer and renewal (9). WM impairment has a direct negative impact on the social life of MDD patients (10). It may weaken their ability to regulate emotions and increase their reflective response to negative events (11). WM impairment can also deepen the loss of pleasure in MDD and further increase the bias of positive self-judgment; it is even considered to be a risk factor for MDD (12, 13). Studies using rat models of depression have shown that WM impairment may occur before depression (14). The operation of WM depends on a series of specific functions, in which interference control basically refers to a person’s ability to effectively keep stimulus, target or background information in an active and accessible state, to effectively suppress the stimulus or response unrelated to the target, or both (15). This ability is considered to be an important component of WM capacity and is applicable to a variety of situations requiring executive control (16, 17). Switching between different tasks or redistributing attention when changing psychological state is responsible for the relationship between each subsystem and their long-term memory, the coordination of attention resources and the selection (18).

Many studies have reported that MDD patients present impairments in WM with interference control. For example, Seibert and Ellis found that depression patients had a high proportion of task-irrelevant ideas and impaired interference control and that the proportion of these ideas was negatively correlated with task performance (19). Moreover, a study used the visuospatial change detection task to investigate WM function of maintenance, based on performance in trials using the targets only, and the WM function of interference control, based on performance in trials with distractor rectangles in MDD patients. The results indicated that MDD patients displayed generalized impairments on visuospatial WM function of maintenance and interference control (20). However, this behavioral study has not been discussed in combination with verbal WM tasks and event-related potential (ERP) technology. Another study used graph theory to examine the functional connectomic metrics (local and global efficiency) at the whole-brain and large-scale network levels in MDD patients during a WM task, and the results showed decreased integration of the frontoparietal network during a WM task in MDD (21).

Depressed patients cannot effectively eliminate interference information due to the decline in WM. A study of eye-tracking design showed that individuals with high WM capacity performed better in resisting interference than individuals with low WM capacity (15). When the load of WM generates a gradient, in low-order working memory tasks, whether MDD patients with impaired interference control can control the central executive resources to complete the low-order WM tasks is not **clear** (22). Therefore, exploring the mechanism of interference control impairment and specifying the possible performance of MDD under different WM gradients will be of great help to better refine WM training and treatment plans in the future. Additionally, it might contribute to a comprehensive understanding of WM impairment in the pathology of depression.

The Brown-Peterson task (BPT) is a paradigm for measuring working memory and assessing verbal short-term memory and distractions (23). With the increase in the difficulty of the distraction task, the subjects’ retention of the initial stimulus is gradually reduced, which may be because the subjects’ capacity for interference control faces greater challenges and they are unable to suppress the competition of distraction tasks for attention resources more efficiently. Studies have shown that BPT is used to measure verbal working memory systems in different populations (24).

Event-related potential (ERP) is a method used to detect cognitive function. It uses the brain potentials caused by multiple stimuli to reflect changes in the cognitive processes of the brain neurophysiology when subjects perform tasks (25). The paradigm used in this study requires participants to respond accurately and quickly. Compared with other imaging techniques, *ERP can be accurate to the millisecond level* and has the advantage of detection time (26). It can be more objective. In fact, many researchers have used ERP to study changes in interference control in MDD under different WM loads. For example, a selective attention task used the auditory oddball paradigm; participants with high WM capacity, rather than participants with low WM capacity, produced smaller *negative 100 (N1*) amplitudes when resisting distractions, while there was no difference in N1 amplitudes under a simple difficulty level of low distraction (27). This may be because participants with high WM capacity have a faster attention process to interference stimuli than participants with low WM capacity, and participants with high WM capacity have a stronger interference control ability for modulation. At the same time, a cognitive reappraisal study found that under low-order difficult levels, compared with negative reappraisal, the late positive potential (LPP) amplitude of neutral reappraisal was significantly reduced. However, under the high-order difficult level, the moderating effect of this reevaluation disappeared. Whatever the picture type is, the LPP amplitude of the high-order task is less than that of the low-order task. This is because participants have to memorize three more symbols in high-order tasks, which consumes more resources and leads to insufficient resources for simultaneous reassessment. It has been suggested that LPP amplitude not only indicates attention to emotional content and promotes processing but also reflects the task-related motivational processing of stimuli (28). Notably, these studies were conducted in normal populations. A study of MDD found that in the Prose Distraction Task (tapping on access function), the MDD group showed difficulty in trying to suppress the intrusion of irrelevant words in WM, slow reading speed and more errors than the control group. The phenomenon of a slower response was also shown in the Stroop task. *It was found that patients with MDD were unlikely to be able to suppress and delete some irrelevant information* (29). Deficiencies associated with depression during conflict resolution were also reported in another inhibitory control study of flanker tasks (30). Thus far, the exploration of WM with interference control in MDD in non-emotional tasks remains to be improved, especially the need to design a richer level of tasks to increase the gradient of subjects’ response.

Although many previous studies have indicated that MDD patients present impairments in WM, the electrophysiological mechanism of interference control dysfunction in MDD under different gradients has not been elucidated. To date, no studies that used the BPT paradigm combined with ERP technology to measure interference control function in MDD have been reported. Further clarifying the ERP characteristics of the interference control dysfunction of WM in MDD would be helpful in understanding the neural mechanism of MDD. Furthermore, investigating the ERP characteristics of the interference control of the impairments of WM has implications for understanding the etiology and the new therapeutic target in MDD. In this study, WM was measured with a BPT, and all participants, including MDD patients and healthy controls (HCs), were measured with ERPs evoked by a BPT. We hypothesized that interference control should be impaired in MDD patients, and more so with rising task difficulty. The functional impairment in MDD should be repflected in abnormal ERP responses, such as a smaller LPP in the patients vs. controls in high-order tasks. The purpose of this study was to investigate the neural mechanism of the interference control dysfunction of WM in MDD.

## Materials and methods

### Time and setting

The study was conducted at Wuxi Mental Health Center, affiliated to Nanjing Medical University, China, from January 1, 2020 to March 31, 2022. The research program was approved by the Ethics Committee of Wuxi Mental Health Center, affiliated to Nanjing Medical University, China, and carried out in accordance with the Declaration of Helsinki (Reference NO. WXMHCIRB 2021LLky080).

### Participants

All MDD patients were recruited from the Department of Psychiatry of Wuxi Mental Health Center, affiliated with Nanjing Medical University, China, including inpatients and outpatients. The inclusion criteria were as follows: (a) met the criteria of MDDas confirmed by the Structured Clinical Interview for DSM disorders (SCID); (b) age range from 18 to 65 years old; (c) had not taken medication which damaged cognitive function, such as atropine, benzodiazepine etc., for the last two weeks; (d) had normal vision; e) had no diagnosis of alcohol, nicotine, or other substance dependency; (f) had no history of electroconvulsive therapy (ECT) or modified ECT within 6 months prior to recruitment; (g) not suffering from a cerebral organ disease or a serious, unstable physical disease, such as heart disease; (h) not met the criteria of any mental disorders rather than MDD according to DSM. HCs were recruited from local residents through advertising. The inclusion criteria were: (a) age, sex and years of education were matched with depression patients, (b) no history or family history of any mental illness.

We fully communicated with the participants about the cautions and risks of the study and obtained written informed consent. We had also obtained the consent from the patients’ guardian or next of kin, if some depression patients have impaired ability to give consent even though they are clinically stable. All participants received 200.00 Yuan RMB (equals to about 30 US dollars) as compensation for their participation in the study.

### Brown-Peterson task measurements

The Brown-Peterson task is a non-emotional task paradigm with multiple gradients, which has some unique advantages compared to the N-back paradigm or other paradigms with single difficulty of interference difficult levels. Of course, the N-back paradigm can also have different levels of difficulty, usually 1-back or 2-back and sometimes 3-back. The difficulty in the n-back arises from interference by an increasing amount of other stimuli (which also have to be memorized). In the BPT, the difficulty arises from secondary tasks (which are unrelated to the main task) with different levels of difficulty. BPT sets a baseline level starting from no task, and the disturbance difficulty transitions from simple finger tapping to counting and subtracting disturbances, so this paradigm has a richer challenge type. In addition, BPT requires the participant to respond with only two buttons on the mouse, which can detect not only inhibition ability but also proximity function.

**FIGURE 1:**
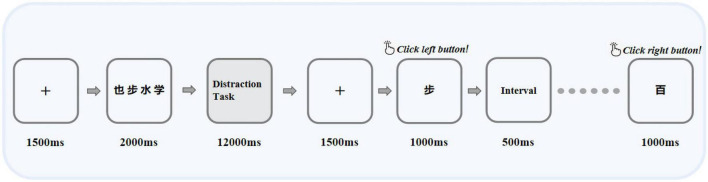
Illustration of the BPT task, in which the interference task includes four difficult levels: no interference task, finger tap, forward counting, and subtraction of three operations. The next difficult level occurred only after each task was completed. “也”, “步”, “水”, and “学” were Chinese characters that appeared in the paradigm, which means “also”, “step”, “water”, and “learn” in English, respectively.

The program design of BPT was similar to a previous study (31). All subjects needed to complete the BPT under four types of distraction tasks (distraction tasks with different difficulty levels below were referred to as difficult levels). These four types of difficult interference levels require different brain processing resources, and the difficulty theoretically increases one by one. There was no difference in the order in which the difficult levels were presented between different subjects. The overall process of each trial was similar. As illustrated in Figure 1, when the trial started, the fixation [“ + “, 1.0 × 1.0 centimeter (cm), lasting *1,500 milliseconds (ms*)] appeared in the center of the screen, and the subject saw four black Chinese characters with a white background. The Chinese characters were only displayed for 2,000 ms, and the subjects were required to memorize them. There was an interval of 12,000 ms after recognition. After the interval was over, eight Chinese characters appeared one by one in the center of the screen, four of them had already appeared, and the other four had not appeared before and needed to be identified. The one that appeared was classified as a target stimulus, named S1, and the one that did not appear was classified as a non-target stimulus, named S2. At the same time, subjects were required to judge the eight Chinese characters appearing as soon as possible on the equipped mouse under the premise of ensuring correctness. They clicked the left button for the four Chinese characters they had known before and clicked the right button for the four Chinese characters they had not seen before. To better adapt the subjects and ensure the accuracy of the data, our recall method chooses to use the mouse to judge instead of verbal or pen writing. The maximum response time of each Chinese character was only 1,500 ms. Chinese characters were selected from the 320 characters most frequently used in the “Modern Chinese Frequency Dictionary” to ensure that the subjects could recognize them. Chinese characters were presented pseudorandomly, with similar word frequencies, no semantic relationship between each other, different shapes and strokes, and little difference in emotional effect.

*In the BPT paradigm*, the same type of difficult level was performed 10 times in succession, and the next type of difficult level was performed after completion. Between each type of difficult level, a 1-min rest period was provided. No interfering work was the first type of difficult level, which was used to determine that the patient could complete the recall task after an interval of 12,000 ms after the memory task. After that, the remaining three types of difficult interference levels were performed in sequence within an interval of 12,000 ms, and the difficulty of each type of level gradually increased compared with the previous level. As inferred, finger tapping is a simple motor skill that should not cause structural dislocation of verbal materials in memory. Studies have shown that pronunciation requires the lowest central processing resources for normal subjects and only causes slight forgetting of the BPT, so forward counting was chosen as a simple pronunciation task. For the most difficult interference difficult level, we chose to subtract three because it consumed the most resources, but it would not be too difficult for the patient to complete the task. In general, the four difficult levels from the lowest to the highest were as follows: (1) Difficult Level 1: no interference difficult level for interruption, the interval of 12,000 ms was just for rest; (2) Difficult Level 2: finger tapping, the subject used the index finger of the dominant hand to tap the tabletop continuously during the interval; (3) Difficult Level 3: count, the count of the number of sounds the subject was asked to make from 1 forward during the interval; (4) Difficult Level 4: subtracting three, the subjects were asked to do a simple continuous subtracting three operation during the interval. The question of the operation was pseudorandom, and each participant needed to answer the same question. Before the formal start of the experiment, all participants needed to practice 10 trials to ensure that they had understood the experiment.

BPT was designed and performed by E-Prime software version 3.0 (Psychology Software Tools, Inc., Sharpsburg, PA, USA). The computer screen used to display all stimuli had a resolution of 1,280 × 1,024, a refresh rate of 60 hertz (Hz), and was 19 inches in size. Participants were seated in a room with moderately reduced light and sound, approximately 60 cm from the screen.

The behavioral indicators to be analyzed mainly include the hit rate of S1 (target stimulus) and its corresponding reaction times (RTs) under each difficult level, which are calculated based on electronic data automatically recorded in E-Prime. To guarantee the validity of the answer, we also calculated the false alarm rate for S2 (non-target stimulus) for each difficult level. If the participant randomly presses the button to obtain a high hit rate in S1, he or she will also get a high false alarm rate in the relative S2.

### EEG recordings and analysis

EEG was continuously recorded by a BioSemi Active Two system with a sampling frequency of 500 Hz. The electrode cap was a customized BrainCap (EasyCap, Herrsching, Germany) containing 64 Ag/AgCl ring electrodes, which were recorded according to the international 10/20 system. Electrodes placed below and above the left eye external angle were used to detect vertical and horizontal electrooculography (EOG). When EEG data were collected, the bandpass filtering of EEG and EOG was 0.05 - 100 Hz, and the electrode impedance was less than 5 kiloohm (kΩ). The reference electrodes were the left and right mastoids, and the ground electrode was placed under the left clavicle.

The recorded EEG data were processed offline using Brain Vision Analyzer 2.0 (Brain Products GmbH, Munich, Germany), and the average value of the left and right mastoids was used as the reference voltage for correction during processing. In each individual trial, the zero phase shift Butterworth filter was used for bandpass filtering between 0.1 and 30 Hz, and the independent component analysis (ICA) algorithm was used to eliminate EOG. After stimulation, the data were segmented from -200 ms to 800 ms. Baseline correction was performed using the average voltage from -200 ms to 0 ms before stimulation. The data with blinking, eye movement, myoelectricity and other artifacts were excluded by the established measurement method. When the voltage gradient of a single channel or a given data segment exceeded 50 microvolts (μV)/ms, the amplitude was ± 75 μV, or the signal was flat (over 100 ms less than 0.5 μV), it was regarded as artifact rejection. The segments with different stimulus markers were averaged in the selected time window.

In the BPT paradigm, during the Chinese character matching stage, the stimulus when the repeated Chinese character appeared was marked as S1, and the stimulus when the non-repeated Chinese character appeared was marked as S2. To calculate the overall average event-related potential response of all subjects to the target stimulus, combined with waveform, topographic map, and various experimental difficulty levels, three different event-related potential components were identified, namely, N100, P200 and LPP. In this study, the N100 amplitude was taken as the average amplitude of Fz, F3, F4, Cz, C3, C4, Pz, P3, P4, Oz, O1, and O2 electrode sites between 70 ms and 110 ms. The electrode positions of P200 were the same as those of N100, and the average amplitude was calculated between 150 ms and 250 ms. *Since LPP may be more centrally distributed, we focused on the centroparietal electrode sites Cz, CPZ, and Pz (32, 33) between 400 ms and 700 ms.*

### Statistical analysis

Quantitative data between the MDD group and HC group were compared using the independent *t* test (two-tailed) and Pearson chi-square test. ANOVA was performed on both behavioral data and event-related potential measurement data, with a confidence level of 5%, one of which was in the difficult level factor (interference difficult level, no interference difficult level vs. Finger tapping vs. Count vs. Subtracting three) and one in the Group factor (group, major depressive disorder vs. HCs). Effect sizes were estimated using η*^2^* and Cohen’s *d*, and the Greenhouse-Geiser method was used to correct the degrees of freedom for the F ratio. *When the interaction was significant, post hoc analysis was performed, and Bonferroni correction was used to control the type I error that may be caused by multiple comparisons.* Pearson correlation analysis was performed between the mean amplitude of LPP and HAMD-24 scores, and it *was also performed on the average amplitude of LPP between the hit rate and RTs.* SPSS 22.0 statistical software was used for statistical analysis of the above data (SPSS, IBM Corp, USA).

## Results

### Demographic characteristics of participants

After excluding incomplete or low-quality data, 44 MDD patient and 44 HC data were retained for analysis. There was no significant difference in the average age, educational level, or gender ratio between the two groups. For depressed patients, the mean fluoxetine-equivalent dose was 34.8 ± 1.1 mg/d, as calculated according to a previous study (34).

The demographic characteristics and clinical information of the two groups are shown in Table 1.

**TABLE 1:** Demographic characteristics and clinical information of participants [mean (SD)].

Variable	MDD (*n* = 44)	HC (*n* = 44)	Test statistic
Age (year)	39.1 (11.1)	39.1 (4.8)	*t* = 0.037, *p* = 0.970
Age range	19-59	28-52	-
Sex (M/F)	19/25	23/21	χ*^2^* = 0.729, *p* = 0.393
Education (years)	13.6 (2.5)	14.4 (3.0)	*t* = –1.405, *p* = 0.164
HAMD-24	14.8 (9.4)	-	-
Medicine (E/M/V/D/S/F)	10/3/5/6/10/5/5	-	-

HC, healthy control; SD, standard deviation; HAMD-24, Hamilton depression scale (24-item edition); Medicine (E: Escitalopram; M: Mianserin; V: Venlafaxine; D: Duloxetine; S: Sertraline; F: Fluoxetine).

### Analysis of behavioral data

With accuracy and RTs as dependent variables, a 2 × 4 repeated-measures ANOVA was performed on behavioral results. Group (MDD group vs. HC group) was used as the between-subjects factor, and difficulty level (Difficult Level 1 vs. Difficult Level 2 vs. Difficult Level 3 vs. Difficult Level 4) was used as the within-subject factor.

### Accuracy and reaction times

**FIGURE 2:**
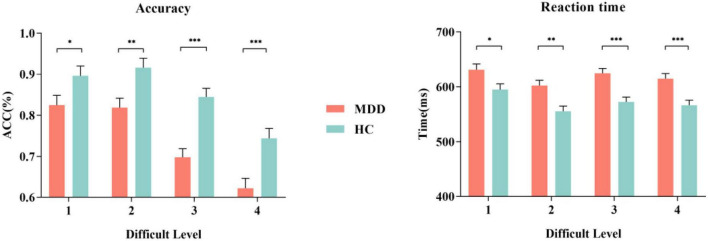
The accuracy and RT of MDD group and HC group for S1. MDD, major depressive disorder; HC, healthy control; ACC, accuracy. *p < 0.050, ** *p* < 0.010, and *** *p* < 0.001.

The mean hit rate and false alarm rate for S1 and S2 under four difficult levels are shown in Figure 2 and Table 2. For the hit rate of S1, repeated-measures ANOVA showed that the interaction for Group × Difficult Level was significant (*F_3,84_* = 2.729, *p* = 0.049, η*2 p* = 0.089), and *the simple effects of groups become obviously larger with rising task difficulty. At each difficult level, the hit rate of the MDD group was significantly lower than that of the HC group.* In the MDD group, the simple effect of difficult level was significant (*F_3,84_* = 28.628, *p* < 0.001, η*2 p* = 0.506). The hit rate of difficult level 4 was the lowest, followed by difficult level 3. Difficult Level 3 and difficult level 4 were significantly different from other difficult levels (*p* < 0.001), *while the same was true for the HC group, and the simple effect in the HC group of difficult level was also significant* (*F_3,84_* = 16.601, *p* < 0.001, η*2 p* = 0.372).

**TABLE 2:** Behavior data [mean (SD)] of HC group (n = 44) and MDD group (*n* = 44) in BPT of 4 difficult levels.

Variables	Difficult level 1		Difficult level 2		Difficult level 3		Difficult level 4	
				
	HC	MDD	HC	MDD	HC	MDD	HC	MDD
Hit rate of S1	0.90 (0.02)	0.83 (0.02)	0.92 (0.02)	0.82 (0.02)	0.85 (0.02)	0.70 (0.02)	0.74 (0.02)	0.62 (0.02)
False Alarm rate of S2	0.06 (0.02)	0.12 (0.02)	0.06 (0.02)	0.13 (0.02)	0.10 (0.02)	0.21 (0.02)	0.11 (0.02)	0.25 (0.03)
RTs for Hit of S1	595.18 (10.42)	631.46 (10.42)	555.59 (9.50)	602.59 (9.50)	572.61 (8.71)	624.90 (8.71)	566.52 (9.27)	615.15 (9.27)
RTs for False Alarm of S2	616.15 (10.68)	657.12 (10.68)	597.47 (10.52)	645.82 (10.52)	612.19 (9.75)	662.37 (9.75)	624.52 (9.82)	659.82 (9.82)

HC, healthy control; MDD, major depressive disorder; RT, reaction time; SD, standard deviation.

For the false alarm rate of S2, repeated-measures ANOVA showed that the interaction for Group × Difficult Level was significant (*F_3,84_* = 3.541, *p* = 0.018, η*2 p* = 0.112), and the simple effects of groups were significant. At each difficult level, the false alarm rate of the MDD group was significantly higher than that of the HC group, and the difference between difficult level 3 and difficult level 4 was the largest. In the MDD group, the simple effect of difficult level was significant (*F_3,84_* = 21.041, *p* < 0.001, η*2 p* = 0.429). The false alarm rate of difficult level 4 was the highest, followed by difficult level 3. Both difficult level 3 and difficult level 4 were significantly different from other difficult levels (*p* < 0.01), *while there was no such difference in the HC group, and the simple effect in the HC group of difficult level was also significant* (*F_3,84_* = 4.360, *p* = 0.007, η*2 p* = 0.135). In general, the hit rate to target stimuli in the two groups under the four difficult levels was high, while the corresponding false alarm rate to non-target stimuli was low, indicating that no participant pressed the space key continuously (or never) during the task.

The RTs of the two groups for S1 and S2 under four difficult levels are shown in Table 2 and Figure 2. For S1 RT, repeated-measures ANOVA showed that the interaction for Group × Difficult Level was not significant (*F_3,84_* = 2.735, *p* = 0.610, η*2 p* = 0.021), and the main effect of Group was significant (*F_3,84_* = 17.948, *p* < 0.001, η*2 p* = 0.391), indicating that the RTs of the MDD group to target stimuli were longer than those of the HC group. For the RTs of S2, repeated-measures ANOVA showed that the interaction for Group × Difficult Level was not significant (*F_3,84_* = 1.341, *p* = 0.267, η*2 p* = 0.046), and the main effects of Group were significant (*F_3,84_* = 7.981, *p* < 0.001, η*2 p* = 0.222), indicating that the RTs of the MDD group to non-target stimuli were still longer than those of the HC group.

### Analysis of event-related potentials data

For the MDD group, the average number of trials for ERP analysis of S1 from difficult level 1 to difficult level 4 was 35.77 ± 4.65, 32.00 ± 6.57, 32.87 ± 6.23 and 33.03 ± 6.45, and those of S2 were 36.00 ± 4.70, 33.14 ± 6.47, 32.48 ± 6.59 and 33.17 ± 6.74. For the HC group, the average number of trials for ERP analysis of S1 was 35.03 ± 4.41, 32.00 ± 4.22, 33.67 ± 5.45, and 33.72 ± 6.14, and those of S2 were 36.06 ± 4.94, 34.72 ± 4.27, 34.22 ± 5.39, and 34.36 ± 6.33.

Using N100, P200, and LPP as dependent variables, a 2 × 4 repeated-measures ANOVA was performed on the mean amplitude, with Group (MDD group vs. HC group) as the between-subjects factor and Difficult Level (Difficult Level 1 vs. Difficult Level 2 vs. Difficult Level 3 vs. Difficult Level 4) as a within-subjects factor.

### N100

For the average amplitude of S1 (target stimuli), the interaction for Group × Difficult Level was not significant (*F_3,258_* = 0.065, *p* = 0.973, η*2 p* = 0.001). For the average amplitude of S2 (non-target stimuli), the interaction for Group × Difficult Level was still not significant (*F_3,84_* = 0.874, *p* = 0.458, η*2 p* = 0.030).

### P200

For the average amplitude of S1, the interaction for Group × Difficult Level was not significant (*F_3,258_* = 1.458, *p* = 0.228, η*2 p* = 0.017). For the average amplitude of S2, the interaction for Group × Difficult Level was still not significant (*F_3,84_* = 1.148, *p* = 0.931, η*2 p* = 0.005).

### Late positive potential

For the mean amplitude of S1, the interaction between difficult level and group was significant (*F_3,84_* = 3.002, *p* = 0.034, η*2 p* = 0.034). In difficult level 3 and difficult level 4, the average amplitude of the MDD group was significantly smaller than that of the HC group (*p*_*level* 3_ < 0.001, *p*_*level* 4_ < 0.001), but there was no such difference in difficult level 1 and difficult level 2 (*p*_*level* 1_ = 0.240, *p*_*level* 2_ = 0.109). In the simple effect of difficult level, the MDD group was significant (*F_3,84_* = 17.253, *p* < 0.001, η*2 p* = 0.381). The average amplitude of difficult level 4 [(1.79 ± 0.40) μV] was significantly lower than that of difficult level 1 [(4.61 ± 0.43) μV], difficult level 2 [(4.84 ± 0.46) μV] and difficult level 3 [(3.29 ± 0.40) μV]. The average amplitude of difficult level 3 was also significantly different from the other three difficult levels. The difficult level simple effect of the HC group was also significant (*F_3,84_* = 5.119, *p* = 0.003, η*2 p* = 0.155). The average amplitude of difficult level 4 [(4.14 ± 0.40) μV] was significantly smaller than that of difficult level 2 [(5.88 ± 0.46) μV] and difficult level 3 [(5.33 ± 0.40) μV].

**FIGURE 3:**
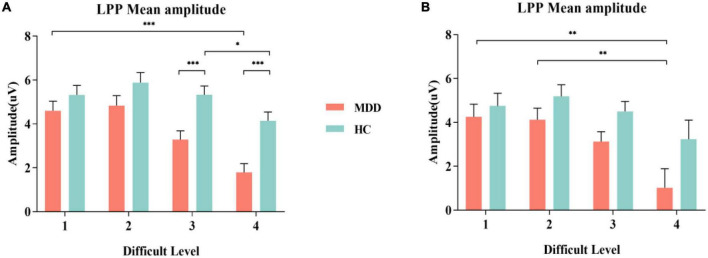
MDD group and HC group were compared in the experiment on the grand average of the LPP mean amplitudes measured at Cz, CPz and Pz electrodes. **(A)** Comparison of S1; **(B)** Comparison of S2. MDD, major depressive disorder; HC, healthy control, LPP, late positive potential. *p < 0.050, ** *p* < 0.010, and *** *p* < 0.001.

**FIGURE 4:**
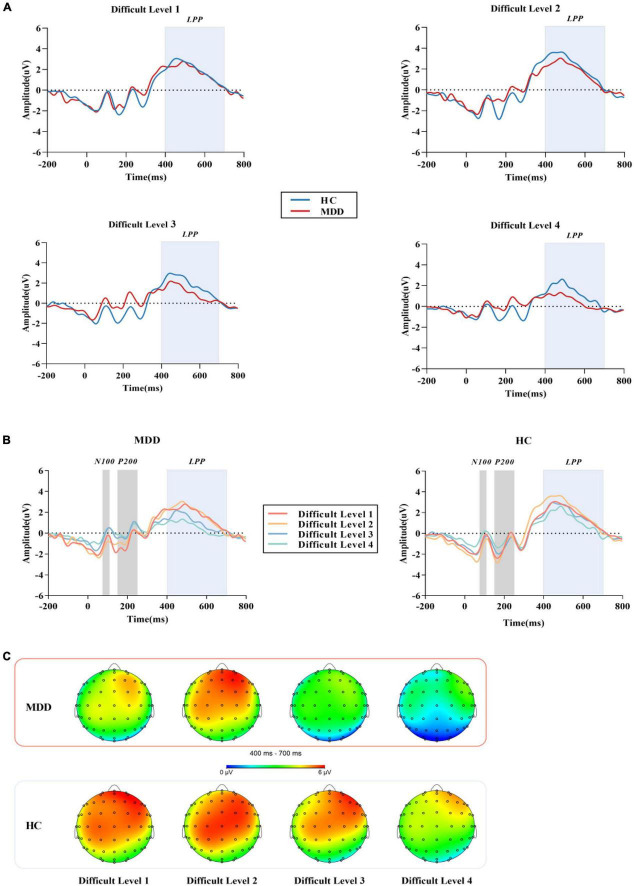
**(A)** The grand average ERP waveforms averaged at Cz, CPz and Pzelectrodes, showing the LPP component of MDD group and HC group in four difficult levels. **(B)** After the stimulation under the four difficult levels started, N100 was measured in the time window of 70 -110 ms, P200 was measured in the time window of 150 - 250 ms, and LPP was measured in the time window of 400-700 ms. **(C)** Topographic distribution of LPP in MDD group and HC group. MDD, major depressive disorder; HC, healthy control; LPP, late positive potential.

For the average amplitude of S2, the main effect of group was significant (*F_1,86_* = 4.402, *p* = 0.039, η*2 p* = 0.049). The average amplitude of the MDD group [(3.13 ± 0.44) μV] was significantly lower than that of the HC group [(4.42 ± 0.44) μV]. The main effect of difficult level was significant (*F_3,84_* = 5.314, *p* = 0.002,η*2 p* = 0.160), and the average amplitude of difficult level 4 [(2.13 ± 0.61)μV] was the smallest. The interaction between difficult level and group was not significant (*F_3,84_* = 0.755, *p* = 0.522, η*2 p* = 0.026) (Figures 3, 4).

*In summary, for both of the average amplitude of S1 and S2, there were all significant differences in different difficult levels between MDD group and HC group.*

### Correlation analysis between late positive potential mean amplitude and HAMD

Pearson correlation analysis was performed on the average amplitude of LPP and HAMD total scores, and no significant correlation was found between any two parameters.

### Correlation analysis between LPP mean amplitude and hit rate and RTs

Pearson correlation analysis was performed on the average amplitude of LPP between the hit rate of S1 and RTs for the hit of S1 from difficult level 1 to difficult level 4 in the two groups, and no significant correlation was found between any two parameters.

## Discussion

In this study, we used the BPT to explore the interference control function of WM and examined whether the verbal WM function of MDD was impaired from the perspective of neuroelectrophysiology. Our findings suggest that even in non-emotional tasks, the MDD group had difficulty suppressing information not related to the WM task. This impairment was not significant in simple WM tasks. Compared with other paradigms, the difficulty of BPT is divided into four gradients. In MDD, the amplitude of LPP was significantly reduced in the high-order task of counting and subtracting, while there was no significant difference in the simple task without distraction or finger tapping. It was suggested that there is a certain degree of interference control impairment in MDD, and the abnormality is particularly significant when the load task challenge becomes larger, and this interference control function impairment may be an important neural correlative factor of abnormal WM in MDD.

The results of behavioral data in this study showed that the accuracy of answers in the depression group under four working memory gradients was significantly lower than that in the HC group (35), and the reaction time was longer (36). Comparison within the MDD group revealed no significant difference between the first two simple distraction *difficulty levels* (37), and there was a significant difference in forward count and minus three tasks. However, combined with the ERP results, we found that there was no significant difference in the performance between the two groups in difficult level 1 and difficult level 2. These results indicated that the MDD group only had lower processing ability for high-order tasks than the HC group. This may be due to the existence of negative cognition in MDD (38). *When they are in the same simple task as others, even if they have the ability to fully resist interference, they will not be able to complete the task normally due to the loss of confidence.* Moreover, after MDD produced incorrect behavioral responses, they were more likely to overexplain the negative information in their attention, it was difficult to eliminate negative materials, and there was a defect in cognitive control when dealing with negative information, resulting in the inability to effectively adjust the psychological state for the next answer. This result is consistent with the research conclusion of Gotlib (39).

Previous studies have shown that N100 and P200 are exogenous components that are affected by physical stimulation, and LPP is an endogenous component (40, 41) that is more related to the subject’s mental state and attention (42). In our ERP results, the average amplitudes of N100 and P200 produced by the target stimuli in the MDD group were not significantly different from those in the HC group, while the LPP was significantly different from that in the HC group. This shows that the MDD group did not receive large external interference in the process of completing the response. Due to the internal interference of the *WM impairment* of the MDD group itself, the results were different (43).

According to previous literature, LPP represents advanced cognitive functions such as refresh (44), retelling and emotional processing of memory, while refresh and retelling depend on the key function named interference control (45). Effectively suppressing and deleting the intrusion of irrelevant information and correctly processing the target stimulus are the key processes for the successful execution of WM (46). In the ERP study of WM function in interference control in MDD and HC groups, previous studies have found that MDD patients have inhibitory deficits both at input levels and transfer levels of emotional information processing (47). Under the premise that the emotions of the target stimuli in this study maintain a neutral balance, the LPP amplitudes generated by MDD when performing difficult level 1 of the no-interference task and the difficult level 2 of finger tapping did not differ significantly from those in the HC group, suggesting that MDD does not significantly damage the input level of non-emotional information. When the difficulty of interfering factors increased, the forward counting task and calculation task in the HC group induced larger LPP amplitudes than those in the MDD group. This may be caused by the reduced flexibility in dealing with irrelevant tasks of the MDD group, and they need to consume more attention resources when resisting high-order interference, so they cannot induce the same amplitude response when confronting the target stimuli, which is also attributed to the reduced cognitive control of distraction processing. This result agreed with previous research findings. Compared with the HC group, WM tasks with the same load are more difficult for low WM capacity people to complete, such as MDD, thus inducing a smaller LPP amplitude (28, 48). When non-target stimuli were presented, behavioral differences were found in S2, but no differences in ERP were found. This might be because S2 did not have the same working memory processing as S1, and the processing of the same group under the same difficulty level was similar. On the other hand, MDD has interference control impairment, and the brain preferentially allocates attention resources to target stimuli, so the results in ERP made a difference (49), but this needs to be further verified. In our study, LPP amplitude was not correlated with HAMD scores, which suggest there could be a dissociation between physiological and psychopathological indices. This is in line with our recent findings in another study (32). In addition, results of a previous study in this field could provide indirect evidence for the interpretation of the present study. In their study, Simone et al. used the HAMD scale to evaluate the psychopathology of MDD, and at the same time assessed WM in cognitive function, and found that psychopathological symptomscan be dissociatedin their impact on cognitive functioning. Our results together with previous findings indicate that LPP amplitude would have the potential to be a trait marker of MDD (50).

Our findings may have reference significance for formulating WM training programs and improving fluid intelligence in MDD (51). In fact, it is impossible for people to complete a task without being disturbed. When the interference is greater, the corresponding ability to adjust and restore is greater. Successful redistribution of attention to major tasks plays a key role in regulating the impact of interference in confrontations with different interference stimuli. A brief rest or learning about the types and knowledge of interference stimuli has a positive effect on enhancing the correct response to impaired interference control (52).

There are still some shortcomings in this study. *Firstly, the inclusion criteria of the age range were relatively large in the present study, which would be a confounder to the results. Therefore, it is warranted to narrow the age range in further studies to obtain more robust findings.* In addition, the sample size of this study was small, thus a larger sample size and the same ERP parameters are required to verify the results. Finally, the results of the study are still insufficient in terms of spatial resolution. The electrophysiological changes generated by different brain regions may be expanded by functional magnetic resonance and other methods, thus helping to further study the neural mechanism of working memory deficits in MDD patients.

In summary, this study shows that the interference control function of WM in MDD is impaired by using the BPT paradigm and ERP technology. In a high-order non-emotional WM task, MDD patients have greater differences and smaller LPP amplitudes than healthy people. Our results provide new evidence for the characteristics and potential neural mechanisms of WM impairment in clinically diagnosed MDD patients, which could help inform more personalized treatment approaches.

## Data availability statement

The original data supporting the conclusions of this article are available from authors upon request.

## Ethics statement

The research program was approved by the Ethics Committee of Wuxi Mental Health Center, affiliated to Nanjing Medical University, China. The patients/participants provided their written informed consent to participate in this study.

## Author contributions

Z-HZ and JW conceived the research, designed the BPT paradigm and revised the manuscript. S-YJ and Z-HZ wrote the manuscript. S-YJ performed the analysis and statistics on participants’ demographic characteristics, BPT behavioral data, and ERP data. S-YJ, R-HS, J-ZZ, and C-GJ oversee data collection and completeness. Z-HZ and JW were responsible for the design and conduct of this study and contributed to data interpretation. All authors contributed to the article and approved the submitted version.
